# Korean survey data reveals an association of chronic laryngitis with tinnitus in men

**DOI:** 10.1371/journal.pone.0191148

**Published:** 2018-01-11

**Authors:** Myung Jin Ban, Won Shik Kim, Ki Nam Park, Jae Wook Kim, Seung Won Lee, Kyungdo Han, Jae Won Chang, Hyung Kwon Byeon, Yoon Woo Koh, Jae Hong Park

**Affiliations:** 1 Department of Otolaryngology-Head and Neck Surgery, Soonchunhyang University College of Medicine, Cheonan, Republic of Korea; 2 Department of Medicine, Graduate School, Yonsei University, Seoul, Republic of Korea; 3 Department of Otorhinolaryngology, Yonsei University College of Medicine, Seoul, Korea; 4 Department of Otolaryngology-Head and Neck Surgery, Soonchunhyang University College of Medicine, Bucheon, Republic of Korea; 5 Department of Otolaryngology-Head and Neck Surgery, Soonchunhyang University College of Medicine, Seoul, Republic of Korea; 6 Department of Biostatistics, College of Medicine, The Catholic University of Korea, Seoul, Republic of Korea; 7 Department of Otorhinolaryngology, Chungnam National University College of Medicine, Daejeon, Korea; International Nutrition Inc, UNITED STATES

## Abstract

The association between chronic laryngitis and tinnitus is not a well-studied topic, unlike the association of these two conditions with many other disorders. Cross-sectional data of 11,347 adults (males: 4,934; females: 6,413), who completed the Korea National Health and Nutrition Examination Survey (KNHANES) from 2010 to 2012 were used to investigate this association. Lifestyle patterns, including smoking and alcohol habits, regular exercise, physical and mental health status, socioeconomic status, nutritional status, and other chronic diseases, were analyzed. Chronic laryngitis and tinnitus were diagnosed by field survey teams, which included otolaryngologists, who conducted chronic disease surveillance using a health status interview, a nutritional status questionnaire, and a physical examination. Chronic laryngitis was significantly associated with age, education beyond high school, depressed mood, voice change, metabolic syndrome, and tinnitus in men. In women, chronic laryngitis was associated with body mass index and diabetes mellitus. Chronic laryngitis in men was significantly associated with tinnitus (odds ratio 1.671, [95% confidence interval: 1.167–2.393]) after adjusting for age, body mass index, smoking status, alcohol intake, regular exercise, metabolic syndrome, education beyond high school, and depressed mood. Additionally, the prevalence of chronic laryngitis increased with increasing severity of tinnitus in men alone (P = 0.002). The study revealed a significant association between chronic laryngitis and tinnitus.

## Introduction

Chronic non-specific laryngitis (CL) is typified by hoarseness and changes of the larynx, such as erythematous swollen mucosa. The yearly incidence of CL in the United States is 3.47 per 1,000 individuals, and up to 21% of the population may develop CL in their lifetime [1]. Smoking and gastroesophageal reflux are the main causes of this disease [2]. Recently, reflux symptoms, psychological stress, and sleep disturbance have also been associated with this condition [3–5]. Usually, CL involves repeated bouts of deterioration and improvement of symptoms over time. Patients with CL experience persistent or episodic symptoms, including a sore throat, globus sensation, hoarse voice, odynophagia, and cough [6].

Tinnitus is a distressing symptom in which unorganized noise is perceived regardless of direction or arises in the head. Subjective tinnitus, which is similar to acoustic hallucinations, is the perception of sound that is not actually present. The pathogenesis of this disorder is not completely understood, and there is no objective test available to detect tinnitus.

Tinnitus occurs in 30–40% of the adult population, and 0.5–2.5% of affected patients suffer from significant discomfort, which may result in poor quality of life [7]. The causative factors of tinnitus are varied. Tinnitus is associated with various psychological disorders, including anxiety, depression, and other conditions involving mental distress [8]. Recent studies have shown that sleep disturbance is also associated with chronic tinnitus [9]. Tinnitus is easily identified by questioning, and its possible origin, which may be a hidden psychological problem, can be determined.

Many previous studies have reported that both CL and tinnitus are commonly associated with mental distress, disturbed sleep, and various psychological and psychiatric disorders. However, the association between CL and tinnitus has not been well studied. For some otolaryngologists, it is of major interest to discern which patients would respond to visceral pain modulators (i.e., tricyclic antidepressants [TCAs] or selective serotonin reuptake inhibitors [SSRIs]) that relieve the perception of reflux symptoms for refractory patients after proton pump inhibitor treatment [10].

The neuroanatomical and physiological association between CL and tinnitus remains unclear. However, a specialized treatment of tinnitus sheds light on a possible association between CL and tinnitus. Transcutaneous vagus nerve stimulation (tVNS), targeting maladaptive neuronal plasticity of the central auditory pathway, which is the cause of tinnitus, is known as one of the treatments for intractable tinnitus, which also reduces sympathetic preponderance [11,12]. Common side effects associated with this treatment are voice changes and coughing; these occur owing to innervation of the recurrent and superior laryngeal nerve branch of the vagus nerve [13]. In patients with tinnitus or depression, sympathetic hyperreactivity due to a stress response is common, suggesting that negative feedback to maintain homeostasis may worsen the common symptoms of laryngitis when the vagus nerve, associated with the parasympathetic nervous system, is activated.

We therefore investigated the association between CL and tinnitus in the South Korean population by analyzing data from the 2010–2012 Korea National Health and Nutrition Examination Survey (KNHANES).

## Methods

### Study population

This study utilized data from the KNHANES, which was performed by the Korea Centers for Disease Control and Prevention from 2010 to 2012. Field survey teams, each including an otolaryngologist, ophthalmologist, and nurses, travelled nationwide with specially equipped mobile examination units. Chronic disease surveillance programs were conducted through use of a health and nutritional status questionnaire, and physical examination. Detailed methodology of the KNHANES has been described in other studies [14–16].

The study enrolled 11,347 participants (male: 4,934, female: 6,413) aged 19 years and older. The study protocol was approved by the Institutional Review Board of the Korea Centers for Disease Control and Prevention (IRB No. 2010-02CON-21-C, 2011-02CON-06-C, and 2012-01EXP-01-2C). Written informed consent was obtained from all study participants prior to commencement of the survey.

### Survey for chronic laryngitis, tinnitus, and life style factors

A survey for CL was conducted based on the diagnostic protocol prepared by the Epidemiologic Survey Committee of the Korean Otolaryngological Society. Each participant who provided a positive response to a questionnaire item asking whether the participant has experienced voice problems was further screened for CL. These participants were then asked about the duration of their symptoms: “How long have you had this voice problem?” The participants could respond with either “> 3 weeks” or “< 3 weeks.” A laryngeal examination, using a 4-mm, 70°-angled rigid endoscope with a charge-coupled device camera, was performed as described previously [14,15].

In order to assess the presence of tinnitus symptoms, participants were asked the question: “Within the past year, did you ever hear a sound (buzzing, hissing, ringing, humming, roaring, machinery noise) originating in your ear?” Participants who answered “yes” to this question were also asked what effect this had on their life in the next question: “How annoying is this noise in your daily life?” (“not annoying,” “annoying [irritating],” “severely annoying, and causes sleep problems”). The participants was answering “annoying” or “severely annoying” were as assigned to the tinnitus group in this study.

Information regarding medical and social history was investigated using self-reported questionnaires, as described previously [14]. Participants were divided into the following three groups for smoking history: “current smoker,” “ex-smoker,” and “non-smoker.” Subjects who consumed alcohol more than once per month during the past year were considered as “regular drinkers.” “Regular exercise” was defined as intense physical activity performed for at least 20 minutes, at least 3 times per week. Participants who had a job were defined as being “employed,” and those who had a life partner were designated as having a “spouse.” Residency was categorized into “urban” and “rural” according to the official address of participants. The category “low income” corresponded to the lowest quartile of annual household income. Education level was classified as high if the participant had completed a qualification beyond high school (ELhsg). Physical and mental health status were classified according to the levels of perceived stress (“light or no stress,” “some or heavy”), the experience of feeling depressed for at least 2 weeks (“yes”, “no”), and suicidal ideation in the last 12 months (“yes”, “no”).

### Anthropometric and laboratory measurements

Weight, waist circumference (WC), and height were measured by nurses, as described previously [14]. Height was measured to the nearest tenth of a centimeter using a SECA 225 (SECA, Hamburg, Germany). Waist circumference (WC) was measured at the level of the midpoint between the iliac crest and the costal margin at the end of normal expiration to the nearest tenth of a centimeter. Weight was measured using a GL-6000–20 scale (Cass, Seoul, Korea) to the nearest tenth of a kilogram. Body mass index (BMI) was calculated as weight (kg)/height squared (m^2^). General obesity was defined as BMI ≥ 25 kg/m^2^ [17,18].

Blood pressure (BP) was measured in a sitting position after 5 min rest. Systolic blood pressure (SBP) and diastolic blood pressure (DBP) were measured on the right upper arm using a mercury sphygmomanometer (Baumanometer; W.A. Baum Co., Copiague, NY). To evaluate the serum levels of biochemical markers, including vitamin D, fasting blood sugar, total cholesterol, triglycerides (TG), high-density lipoprotein (HDL) cholesterol, and low-density lipoprotein (LDL) cholesterol, blood samples were obtained from the antecubital veins of the subjects after overnight (10–12 h) fasting as described previously [14]. Serum levels of biochemical markers were measured using an enzymatic method (Hitachi Automatic Analyzer 7600; Hitachi, Tokyo, Japan).

### Audiometric measurement

Pure-tone audiometric tests were performed using an SA 203 audiometer (Entomed, Malmö, Sweden) at the soundproofing facility of the vehicle reserved for the KNHANES, as described previously [19]. The automated hearing test was conducted at the frequency ranges of 0.5, 1, 2, 3, 4, and 6 kHz.

### Definitions of chronic laryngitis, metabolic syndrome, and hearing loss

CL was diagnosed by laryngoscopic examination in cases of chronic laryngeal inflammation with erythema, edema, pseudosulcus, Reinke’s edema, or thick endolaryngeal mucus. Two otolaryngologists from the Korean Society of Otorhinolaryngology-Head and Neck Surgery verified the recorded documentation, which was obtained as 640 × 480-sized audio–video interleave files compressed by DivX 4.12 codec using a compression rate of 6 Mb/s.

To define the metabolic syndrome (MetS), we followed the criteria set out by the American Heart Association and the National Heart, Lung, and Blood Institute together with the International Diabetes Federation in 2009 [20][17]. MetS was diagnosed when at least three of the following criteria were met: (1) WC ≥ 90 cm in men and ≥ 80 cm in women, according to the International Diabetes Federation criteria for Asian countries; (2) fasting blood sugar ≥ 100 mg/dL or use of medication for elevated glucose; (3) fasting triglyceride ≥ 150 mg/dL or use of cholesterol-lowering medication; (4) HDL cholesterol < 40 mg/dL in men and < 50 mg/dL in women or use of cholesterol-lowering medication; and (5) SBP ≥ 130 mmHg and/or DBP ≥ 85 mmHg or use of antihypertensive drugs in patients with a history of hypertension.

We defined participants with hearing loss as those who had a threshold of amplitude exceeding 40 decibel, averaged at frequencies of 0.5, 1, 2, and 4 kHz, in the ear that had the lower hearing function.

### Statistical analysis

The nationwide data were analyzed statistically using the SAS survey procedure (version 9.3; SAS Institute, Cary, NC) to examine the association between CL, tinnitus, and risk factors in a complex sampling design. To reflect nationwide prevalence estimates, sample weights from the KNHANES were applied in all analyses as described previously [14]. All P-values were two-sided, and P < 0.05 was considered statistically significant.

Participants’ characteristics were described using the mean and standard error (SE) for continuous variables, and number and percentage (SE) for categorical variables. Multiple logistic regression analyses were used to test the association between CL and tinnitus. We first adjusted for age and sex (model 1); then, we adjusted for the variables in model 1 plus BMI (model 2); and, finally, for the variables in model 2 plus smoking status, alcohol consumption, regular exercise, MetS, ELhsg and depressed mood (model 3). In addition, the interaction of sex and the association between CL and tinnitus was investigated.

## Results

### General characteristics of the study population

Among the 11,347 adult participants (≥ 19 years of age, male: 4,934, female: 6,413), 344 had CL and 2527 had tinnitus. The baseline health-related characteristics of the participants according to CL and tinnitus status are shown in Table 1. Factors associated with CL and tinnitus is shown for each of the sexes separately in Tables 2 and 3. We studied males and females independently due to the significant interaction of sex with the association between CL and tinnitus (Table 4). When both sexes were combined, age, smoking, level of education, presence of MetS, diabetes, hypertension and voice change were significant associated with CL. Age, vitamin D, smoking, alcohol drinking, employed status, level of education, low income, severe stress, depressed mood, suicidal ideation, presence of MetS, diabetes, hypertension, voice change, and hearing loss were significantly associated with tinnitus in both sexes (Table 1).

**Table 1:** Analysis of potential factors associated with chronic laryngitis and tinnitus.

Parameter	Chronic laryngitis			Tinnitus		
	No (n = 11003)	Yes (n = 344)	P value	No (n = 8820)	Yes (n = 2527)	P value
**Age (years)**	44.9 ± 0.3	49.2 ± 0.9	<0.0001*	44.2 ± 0.3	48.1 ± 0.6	<0.0001*
**Body mass index (kg/m**^**2**^**)**	23.62 ± 0.05	23.93 ± 0.23	0.1764	23.66 ± 0.05	23.51 ± 0.09	0.1135
**Waist circumference (cm)**	81 ± 0.2	82.5 ± 0.8	0.0666	81.1 ± 0.2	80.9 ± 0.3	0.4254
**Vitamin D (ng/mL)**	17.1 ± 0.2	17.2 ± 0.4	0.8461	17.2 ± 0.2	16.6 ± 0.2	0.0005*
**Current smoker (%)**	23.8 (0.6)	34.9 (4.2)	0.0051*	24.8 (0.6)	21.6 (1.1)	0.0200*
**Regular drinker (%)**	58.9 (0.7)	63.4 (3.9)	0.2685	60.5 (0.7)	53.6 (1.4)	<0.0001*
**Routine exercise (%)**	20.3 (0.6)	21.0 (2.4)	0.7434	20.2 (0.6)	20.5 (1.1)	0.8429
**Spouse (%)**	77.8 (0.9)	81.5 (2.6)	0.1888	78.1 (1)	77.3 (1.2)	0.5924
**Job (%)**	64.7 (0.6)	67.9 (3.4)	0.3614	66.3 (0.7)	59.0 (1.3)	< .0001*
**Residential area: urban (%)**	79.1 (2)	85.6 (3.8)	0.1153	79.7 (2)	77.8 (2.4)	0.1641
**Education beyond high school (%)**	71.2 (0.8)	63.6 (3.1)	0.0119*	73.2 (0.8)	62.5 (1.5)	< .0001*
**Income: lower quartile (%)**	16.1 (0.6)	18.7 (2.6)	0.2783	14.5 (0.6)	22.7 (1.3)	< .0001*
**Severe stress (%)**	28.2 (0.6)	28.7 (3)	0.8642	26.9 (0.6)	33.1 (1.1)	< .0001*
**Depressed mood (%)**	13.2 (0.4)	18.1 (3.2)	0.0871	12.1 (0.4)	18.3 (1)	< .0001*
**Suicidal ideation (%)**	14.2 (0.4)	17.9 (3.6)	0.2735	12.5 (0.4)	21.3 (1)	< .0001*
**Presence of metabolic syndrome (%)**	25.1 (0.6)	36.5 (3.8)	0.0012*	24.2 (0.6)	30.6 (1.4)	< .0001*
**Presence of diabetes mellitus (%)**	8 (0.3)	14.1 (2.3)	0.001*	7.7 (0.4)	10.1 (0.8)	0.0034*
**Presence of hypertension (%)**	26.9 (0.6)	34.4 (3.6)	0.0285*	26 (0.6)	31.5 (1.2)	<0.0001*
**Recognition of voice change (%)**	5.8 (0.3)	22.0 (3.3)	<0.0001*	5.3 (0.3)	10.2 (0.8)	<0.0001*
** ≤ 3 weeks (%)**	2.5 (0.2)	7.7 (1.9)	<0.0001*	2.1 (0.2)	4.5 (0.5)	<0.0001*
** > 3 weeks (%)**	3.3 (0.2)	14.2 (2.6)	<0.0001*	3.1 (0.2)	5.6 (0.6)	<0.0001*
**Unilateral hearing loss (> 40 dB, %)**	5.9 (0.3)	1.9 (1.6)	0.1892	4.8 (0.3)	10.6 (0.8)	<0.0001*

Total n = 11,347, Data are expressed as the mean ± SE or the percentage (SE).

*Significant at P < 0.05

**Table 2:** Analysis of factors associated with chronic laryngitis, according to sex.

Parameter	Chronic laryngitis in men			Chronic laryngitis in women		
	No (n = 4747)	Yes (n = 187)	P value	No (n = 6256)	Yes (n = 157)	P value
**Tinnitus (%)**	3.2 (0.5)	5.9 (1.2)	0.0007*	2.3 (0.3)	2.4 (0.5)	0.8332
**Age (years)**	44 ± 0.4	49.2 ± 1.1	<0.0001*	45.7 ± 0.3	49.1 ± 1.6	0.0352*
**Body mass index (kg/m**^**2**^**)**	24.1 ± 0.1	23.8 ± 0.3	0.3166	23.2 ± 0.1	24.1 ± 0.4	0.0230*
**Waist circumference (cm)**	84.2 ± 0.2	84.1 ± 0.8	0.8856	77.8 ± 0.2	79.9 ± 1.4	0.1408
**Vitamin D (ng/mL)**	18 ± 0.2	17.8 ± 0.6	0.6967	16.2 ± 0.2	16.3 ± 0.5	0.9392
**Current smoker (%)**	42.7 (0.9)	51.4 (5.5)	0.1234	5.5 (0.4)	7.9 (2.7)	0.3134
**Regular drinker (%)**	76.6 (0.8)	78 (4.2)	0.7556	41.7 (0.9)	39.7 (5.2)	0.7192
**Routine exercise (%)**	24 (0.8)	19.3 (2.8)	0.1292	16.6 (0.7)	23.9 (4.6)	0.0716
**Spouse (%)**	81.7 (1.1)	88.5 (3.1)	0.0823	74.2 (0.9)	71 (5.1)	0.5229
**Job (%)**	78.4 (0.8)	78 (4.3)	0.9224	51.3 (0.9)	51.6 (5.3)	0.9546
**Residential area: urban (%)**	79 (2)	86.1 (4.1)	0.1232	79.2 (2.1)	85 (4.5)	0.2528
**Education beyond high school (%)**	77.3 (0.9)	68.2 (3.9)	0.0131*	65.3 (1)	56.3 (4.6)	0.0481*
**Income: lower quartile (%)**	14.1 (0.7)	19.3 (3.8)	0.1297	18.2 (0.8)	17.9 (3.2)	0.9372
**Severe stress (%)**	25.4 (0.8)	25.7 (4.1)	0.9559	30.8 (0.8)	33.6 (4.4)	0.5175
**Depressed mood (%)**	9.4 (0.5)	16.5 (4.4)	0.0421*	16.9 (0.6)	20.7 (4.7)	0.3833
**Suicidal ideation (%)**	10 (0.5)	16 (5.3)	0.1762	18.3 (0.6)	20.9 (4.6)	0.5501
**Presence of metabolic syndrome (%)**	25.9 (0.8)	34.9 (4.2)	0.0245*	23.8 (0.7)	41.5 (5.4)	0.0002*
**Presence of diabetes mellitus (%)**	9.2 (0.5)	13.8 (2.9)	0.0657	7 (0.4)	14.4 (3.8)	0.0064*
**Presence of hypertension (%)**	30.5 (0.9)	37.4 (5)	0.1565	23.4 (0.7)	29.5 (4.5)	0.1501
**Recognition of voice change (%)**	4.5 (0.4)	18.4 (3.7)	<0.0001*	7.1 (0.4)	26.9 (4.9)	<0.0001*
**≤ 3 weeks (%)**	1.8 (0.2)	4.6 (1.9)	<0.0001*	3.1 (0.3)	12.8 (3.4)	<0.0001*
**> 3 weeks (%)**	2.6 (0.3)	14.2 (3.6)	<0.0001*	3.9 (0.3)	14.2 (3.3)	<0.0001*
**Unilateral hearing loss (> 40 dB, %)**	6.4 (0.5)	7 (1.9)	0.7595	5.5 (0.4)	9.3 (3.1)	0.1223

Total n = 11,347. Data are expressed as the mean ± SE or the percentage (SE).

*Significant at P < 0.05

**Table 3:** Analysis of factors associated with tinnitus according to sex.

Parameter	Tinnitus in men			Tinnitus in women		
	No (n = 3919)	Yes (n = 1015)	P value	No (n = 4901)	Yes (n = 1512)	P value
**Age (years)**	43.2 ± 0.4	48.7 ± 0.7	<0.0001*	45.2 ± 0.3	47.6 ± 0.7	0.0005*
**Body mass index (kg/m**^**2**^**)**	24.1 ± 0.1	23.9 ± 0.1	0.1014	23.2 ± 0.1	23.2 ± 0.1	0.7988
**Waist circumference (cm)**	84.3 ± 0.2	84.1 ± 0.4	0.7451	77.7 ± 0.2	78.3 ± 0.4	0.1036
**Vitamin D (ng/mL)**	18.1 ± 0.2	17.7 ± 0.3	0.2321	16.4 ± 0.2	15.8 ± 0.2	0.0062*
**Current smoker (%)**	43.5 (1)	40.7 (2.1)	0.2449	5.1 (0.4)	7 (0.8)	0.0249*
**Regular drinker (%)**	77.5 (0.9)	73.1 (1.8)	0.0188*	42.6 (0.9)	38.6 (1.8)	0.037*
**Routine exercise (%)**	23.5 (0.9)	25.5 (1.7)	0.2686	16.8 (0.7)	16.6 (1.4)	0.8828
**Spouse (%)**	81.1 (1.3)	85.8 (1.6)	0.0178*	75.1 (1.1)	70.8 (1.6)	0.0157*
**Job (%)**	79.5 (0.9)	73.4 (1.8)	0.0022*	52.4 (1.0)	47.9 (1.6)	0.0163*
**Residential area: urban (%)**	79.5 (2)	77.9 (2.6)	0.3746	79.8 (2.0)	77.7 (2.5)	0.1978
**Education beyond high school (%)**	78.6 (0.8)	70.1 (1.8)	<0.0001*	67.6 (1.0)	56.7 (1.9)	<0.0001*
**Income: lower quartile (%)**	13.1 (0.7)	19.6 (1.6)	<0.0001*	16 (0.7)	25.1 (1.5)	<0.0001*
**Severe stress (%)**	24.4 (0.8)	30.1 (1.7)	0.0013*	29.5 (0.9)	35.5 (1.5)	0.0003*
**Depressed mood (%)**	8.5 (0.5)	14.6 (1.4)	<0.0001*	15.8 (0.7)	21.1 (1.4)	0.0002*
**Suicidal ideation (%)**	8.9 (0.5)	16.2 (1.4)	<0.0001*	16.2 (0.7)	25.3 (1.4)	<0.0001*
**Presence of metabolic syndrome (%)**	25.5 (0.9)	32.4 (2.3)	0.0024*	22.9 (0.8)	29.1 (1.6)	0.0003*
**Presence of diabetes mellitus (%)**	8.9 (0.5)	11.4 (1.3)	0.045*	6.6 (0.4)	9 (1.0)	0.014*
**Presence of hypertension (%)**	29.5 (0.9)	36.3 (2)	0.0007*	22.3 (0.7)	27.8 (1.4)	<0.0001*
**Recognition of voice change (%)**	4 (0.4)	9.4 (1.1)	<0.0001*	6.6 (0.5)	10.8 (1.0)	<0.0001*
**≤3 weeks (%)**	1.5 (0.2)	3.9 (0.8)	<0.0001*	2.9 (0.3)	5.0 (0.7)	<0.0001*
**>3 weeks (%)**	2.5 (0.3)	5.5 (0.9)	<0.0001*	3.7 (0.4)	5.7 (0.7)	<0.0001*
**Unilateral hearing loss (>40 dB, %)**	5.2 (0.5)	12.2 (1.2)	<0.0001*	4.4 (0.4)	9.4 (0.9)	<0.0001*

Total n = 11,347. Data are expressed as the mean ± SE or the percentage (SE).

*Significant at P < 0.05

**Table 4:** Logistic regression models of tinnitus for chronic laryngitis.

Parameter	Model 1			Model 2			Model 3		
	OR (95% CI)	P-value	P-value for interaction	OR (95% CI)	P-value	P-value for interaction	OR (95% CI)	P-value	P-value for interaction
**Total**	1.383 (1.035–1.848)	0.0284		1.378 (1.032–1.840)	0.0298		1.358 (1.019–1.809)	0.0366	
**Men**	1.683 (1.154–2.453)	0.0068	0.0671	1.664 (1.145–2.418)	0.0075	0.066	1.671 (1.167–2.393)	0.0050	0.0464
**Women**	1.012 (0.654–1.567)	0.9578		1.016 (0.657–1.572)	0.942		0.981 (0.619–1.554)	0.9352	

Individuals without tinnitus were used as the reference category.

OR = odds ratio, CI = confidence interval

Model 1: Adjusted for age and sex. Model 2: Adjusted for age, sex, and body mass index (BMI). Model 3: Adjusted for age, sex, BMI, smoking status, alcohol intake, regular exercise, metabolic syndrome, education beyond high school, and depressed mood.

Sex was an adjusting factor only for the total population

### Factors associated with chronic laryngitis and tinnitus, by sex

Participants, of either sex, with CL were more likely to be older (Table 2). ELhsg, MetS, and the recognition of voice change were significantly associated with CL in both sexes. The prevalence of tinnitus and depressed mood were significantly higher among males with CL than those without CL. Mean BMI and the prevalence of diabetes mellitus were significantly higher among females with CL than those without CL.

Sex-wise factors associated with tinnitus are given in Table 3. Age, alcohol drinking, having a spouse, employed job, ELhsg, low income, severe stress, depressed mood, suicidal ideation, presence of MetS, diabetes, hypertension, voice change, and hearing loss were significantly associated with tinnitus in both sexes. Vitamin D and smoking were significantly associated with tinnitus in women.

Age and ELhsg were significant factors commonly associated with CL and tinnitus in both male and female patients. The prevalence of MetS and voice changes was significantly higher in patients with CL or tinnitus than in those without CL or tinnitus, regardless of symptom duration, in both sexes. The prevalence of depressed mood was significantly higher in men with CL as well as in those with tinnitus (men with CL: 16.5 ± 4.4% versus 9.4 ± 0.5%, P = 0.042; men with tinnitus: 14.6 ± 1.4% versus 8.5 ± 0.5%, P < 0.001).

The high incidence of laryngitis and tinnitus in the elderly was thought to be related to vocal cord atrophy and degeneration of the auditory nerve. The increase in social activity related to the increase of the education level is likely to have decreased the incidence of these diseases or the self-questionnaire response rate [5,6]. The atherosclerosis process accelerated by MetS causes deterioration of tinnitus, while CL, which is caused by laryngopharyngeal reflux in patients with central obesity, which represents MetS, is a common cause of voice change [14,21]. Depressed mood is one of the psychosocial causes of laryngitis and tinnitus, but the predominantly male tendency in men has not previously been reported [22,23].

### Associations between chronic laryngitis and tinnitus

Table 4 shows the degree of association between CL and tinnitus in both sexes after adjusting for confounders by logistic regression models. The adjusted odds ratio (OR) for CL was not increased in women with tinnitus. CL; however, it was significantly associated with tinnitus in men (OR [95% CI]: 1.683 [1.154, 2.453] in model 1, OR [95% CI]: 1.664 [1.145, 2.418] in model 2, and OR [95% CI]: 1.671 [1.167, 2.393] in model 3), after adjusting for confounders.

**Fig 1:**
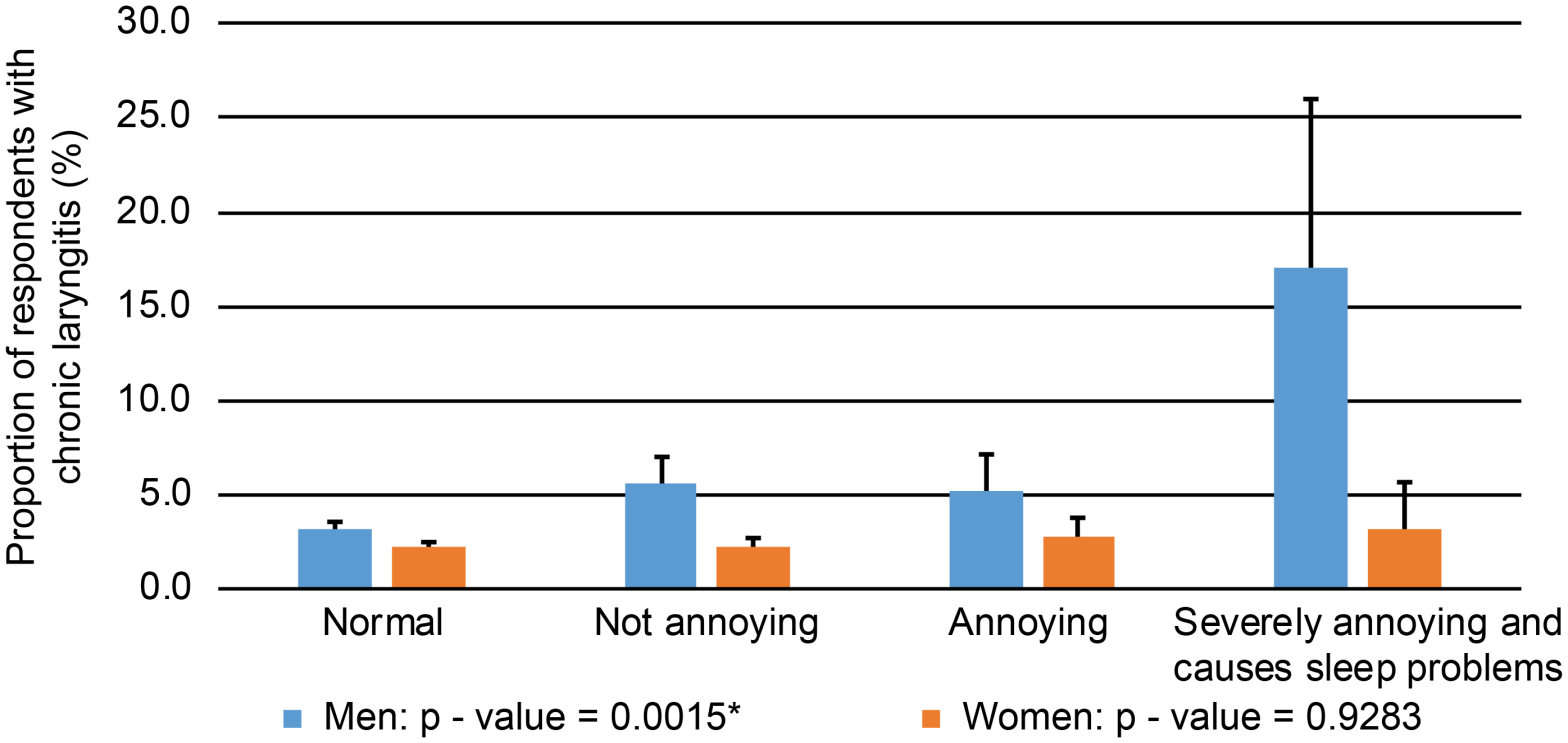
Prevalence of chronic laryngitis according to the severity of tinnitus in men and women.

Each of models 1, 2, and 3 were adjusted for demographic variables only, demographic plus anthropometric variables, and demographic plus anthropometric plus socioeconomic or chronic adult disease variables, respectively. Factors associated with chronic adult diseases were integrated into MetS and were reflected in the logistic model. Age, sex, BMI, smoking status, alcohol intake, regular exercise, MetS, ELhsg, and depressed mood were factors for which adjustments were made in model 3. S1 Table shows the ORs for all the factors considered for model 3. The P-value for interaction between sex and the association between CL and tinnitus showed a significantly different association pattern. Fig 1 shows that the prevalence of CL increased significantly with increasing tinnitus severity in men only (P = 0.002).

## Discussion

CL is a chronic, non-infective inflammatory condition of the laryngeal mucosa, which has a subtle onset and can last for weeks or months [6]. In non-smokers, the most common cause of CL, otherwise known as recurring irritative laryngitis, is gastroesophageal acid reflux, often referred to as laryngopharyngeal reflux (LPR) [3]. LPR is a very common condition, affecting 35% of people over the age of 40 years [24]. Recently, LPR has been reported to be associated with obstructive sleep apnea [25]. A previous study demonstrated a markedly higher prevalence of laryngeal inflammation in people with sleep apnea than in those without a history of sleep disorder [26]. Moreover, a recent study reported positive associations between gastroesophageal reflux disease, sleep disturbances, and psychological stress [22].

Tinnitus has a similarly high lifetime risk of 30–40% in the adult population and has also been associated with sleep disturbances and psychological stress [8,9]. As our cross-sectional study shows, depressed mood was positively associated with CL and tinnitus in men. CL was significantly associated with tinnitus after adjusting for confounders. Similar to these results, psychosocial factors, including disturbed sleep, depression, anxiety, stress, and lifestyle, have been shown to be associated with CL and tinnitus [4,5,8,9,14,25].

Sleep disturbance may affect anxiety and depression, both of which have been positively associated with CL and tinnitus. Further, anxiety and depression can in turn aggravate sleep disturbance. Therefore, sleep disturbance, anxiety, and depression can exacerbate each other’s progression [23,27].

The association between sleep disturbance and tinnitus can be explained based on neurophysiological mechanisms of the limbic system. Wallhäusser-Franke et al. [9] showed that both insomnia patients and animal models of tinnitus have an activated limbic system, indicating that insomnia and tinnitus may share common physiological mechanisms.

As reported in previous studies, psychosocial factors may affect the incidence and course of the disease, not only in CL, but also in tinnitus. In the current study, we demonstrated the various factors associated with CL. In men, age, ELhsg, MetS, depressed mood, voice change, and tinnitus were associated with CL. However, only BMI and DM were associated with CL in women. Our results for several of these parameters differ from those of previous studies [14,21].

To the best of our knowledge, the association between CL and tinnitus based on analysis of a representative nationwide, large population-based dataset, stratified by sex, has not been reported to date. This study reports a correlation pattern that differs by sex. Positive correlations between men with CL and tinnitus in models 1, 2, and 3, after adjusting for age, BMI, smoking status, alcohol intake, regular exercise, MetS, ELhsg, and depressed mood were noted. Voice change was not considered as an adjusting factor, to avoid overcorrection. However, in women, there was no correlation between CL and tinnitus in models 1, 2, or 3 (Table 4). The reason for this sex difference in the relationship between CL and tinnitus is not clear. However, interestingly, depressed mood was significantly associated with CL and tinnitus in men alone, as was the association between CL and tinnitus; however, this was not observed in women. There were significant correlations between depression, CL, and tinnitus in men.

This study has some limitations. First, there was no detailed questionnaire to grade the severity of laryngitis symptoms. However, the questions concerning the severity of tinnitus were more detailed. Second, the data contained several parameters from a self-reported questionnaire, such as smoking, alcohol intake, and income, which may have led to under- or over-reporting by patients. Third, no known biological pathway that could explain this association has been reported in the literature. As described earlier, only psychosocial factors are known to associate with both CL and tinnitus, such as sleep, anxiety, depression, stress, and life style; as shown in Tables 2 and 3, depressed mood was the common associated factor. Although there is no common biological connection, this novel statistical association may facilitate understanding of clinical symptoms or treatment, such as in patients with globus or subjective tinnitus. Globus is a persistent non-painful sensation of a lump in the throat, which is usually long lasting and difficult to treat. Globus patients without laryngopharyngeal reflux, which is the major etiological factor, or those who do not respond to proton pump inhibitors, have significantly more psychological symptoms [28]. Subjective tinnitus is the perception of sound in the absence of auditory stimulation. In terms of neurophysiology, loss of central inhibition in the damaged region, or cortical active plasticity, or neuronal hyperactivity of the central auditory pathway are known pathophysiological factors, but the cause of tinnitus is poorly understood, and it remains difficult to treat [29]. Intractable tinnitus following depression is well known and antidepressants are a useful treatment even in patients who have not had depression or anxiety disorders [30]. Given the association of CL and tinnitus in men in our study, we hypothesize that concurrent CL and tinnitus in men may respond effectively to antidepressants, and should be assessed in future.

This study has a major strength in that it adjusted for a number of covariates and that reliable data were obtained under supervision of an otolaryngologist. In addition, we demonstrated that depressed mood was associated with both CL and tinnitus in men (Tables 2 and 3). No previous studies on associated factors of CL, or of the relationship between CL and tinnitus, in a large representative population have been published. In this study, data were collected from a national survey in South Korea, and analysis showed that the psychosocial factors associated with CL (i.e., age, BMI, ELhsg, DM, MetS, depressive mood, and voice change) differed according to sex, and that CL was associated with tinnitus in men after adjusting for the other confounding factors. Moreover, different patterns of correlation according to sex and the severity of tinnitus were found, and CL prevalence in men increased with tinnitus severity.

## Conclusions

CL was significantly associated with tinnitus in men. We suggest that patients with either of these conditions be screened for the other condition, as well as for other associated factors, such as depressed mood. Further prospective observational studies on the relationship between CL and tinnitus are needed to investigate the potential underlying mechanisms.

## Supporting information

S1 Table Odds ratios for adjusting factors considered for model 3 in Table 4. (DOCX)
