# Supplementary material for: Korean survey data reveals an association of chronic laryngitis with tinnitus in men
Source: PLoS One. 2018 Jan 11;13(1):e0191148. doi: 10.1371/journal.pone.0191148 (PMC5764343; doi:10.1371/journal.pone.0191148)
Supplement: S1 Table — (DOCX) [file pone.0191148.s001.docx]

**S1 Table. Odds ratios for adjusting factors considered for model 3 in Table 4.**

|  | **Total** | | **Male** | **Female** |
| --- | --- | --- | --- | --- |
| **Tinnitus** | 1.358(1.019–1.809) | | 1.671(1.167–2.393) | 0.981(0.619–1.554) |
| **Age** | 1.014(1.002–1.026) | | 1.019(1.005–1.033) | 1.002(0.981–1.024) |
| **Sex (Female)** | 0.659(0.458–0.949) | |  |  |
| **Body mass index (kg/m^2^)** | 0.996(0.953–1.04) | | 0.977(0.92–1.039) | 1.02(0.964–1.08) |
| **Current smoker (%)** | 1.523(1.012–2.293) | | 1.561(0.992–2.458) | 1.511(0.755–3.025) |
| **Regular drinker (%)** | 0.73(0.429–1.244) | | 0.727(0.419–1.26) | 0.879(0.126–6.107) |
| **Routine exercise (%)** | 1.043(0.766–1.422) | | 0.785(0.534–1.156) | 1.617(0.971–2.693) |
| **Metabolic syndrome (%)** | 1.562(1.098–2.222) | | 1.36(0.877–2.109) | 2.208(1.331–3.665) |
| **Education beyond high school (%)** | 1.003(0.666–1.512) | 0.978(0.581–1.646) | | 1.037(0.542–1.986) |
| **Depressed mood (%)** | 1.551(0.994–2.42) | 1.885(0.991–3.587) | | 1.195(0.669–2.137) |

The P-value for interaction was evaluated between men and women (0.0464).
